# Alternative splicing of the *Anopheles gambiae Dscam *gene in diverse *Plasmodium falciparum *infections

**DOI:** 10.1186/1475-2875-10-156

**Published:** 2011-06-08

**Authors:** Paul H Smith, Jonathan M Mwangi, Yaw A Afrane, Guiyun Yan, Darren J Obbard, Lisa C Ranford-Cartwright, Tom J Little

**Affiliations:** 1 Institute of Evolutionary Biology and Centre for Immunity, Infection and Evolution, School of Biological Sciences, University of Edinburgh, EH9 3JT Edinburgh, UK; 2 Institute of Infection, Immunity and Inflammation, College of Medical, Veterinary and Life Sciences, University of Glasgow, G12 8QQ Glasgow, UK; 3 Centre for Vector Biology and Control Research, Kenya Medical Research Institute, Mumias Rd, Kisumu, Kenya; 4 Program in Public Health, University of California at Irvine, Irvine, CA 92697, USA

## Abstract

**Background:**

In insects, including *Anopheles *mosquitoes, Dscam (Down syndrome cell adhesion molecule) appears to be involved in phagocytosis of pathogens, and shows pathogen-specific splice-form expression between divergent pathogen (or parasite) types (e.g. between bacteria and *Plasmodium *or between *Plasmodium berghei *and *Plasmodium falciparum*). Here, data are presented from the first study of *Dscam *expression in response to genetic diversity within a parasite species.

**Methods:**

In independent field and laboratory studies, a measure of Dscam splice-form diversity was compared between mosquitoes fed on blood that was free of *P. falciparum *to mosquitoes exposed to either single or mixed genotype infections of *P. falciparum.*

**Results:**

Significant increases in *Anopheles gambiae *Dscam (AgDscam) receptor diversity were observed in parasite-exposed mosquitoes, but only weak evidence that AgDscam diversity rises further upon exposure to mixed genotype parasite infections was found. Finally, a cluster of *AgDscam *exon 4 variants that become especially common during *Plasmodium *invasion was identified.

**Conclusions:**

While the data clearly indicate that AgDscam diversity increases with *P. falciparum *exposure, they do not suggest that AgDscam diversity rises further in response to increased parasite diversity.

## Background

The innate immune system is common to both invertebrates and vertebrates, and although less well studied than the adaptive immune response, is probably responsible for eliminating the majority of infectious organisms. This is achieved through engulfing cells (e.g. phagocytes), antimicrobial compounds (e.g. defensins) and non-specific reactive intermediates such as nitric oxide [1-5]. The vertebrate adaptive immune system appears to have more complex features: functional antibodies assembled by V-(D)-J joining of gene segments and diversified by somatic hypermutation accommodate an unrivalled resolution in terms of pathogen recognition for the vertebrate adaptive immune response [6]. Invertebrates, lacking antibodies and having only an innate immune system, rely on germline encoded pattern recognition receptors (PRRs) to detect pattern associated molecular patterns and initiate a response [2,7-9].

The absence of an equivalent of the vertebrate adaptive immune system has long fostered doubts that the invertebrate immune system could incorporate specificity and/or memory [10-12]. However, the absence of the cellular and genetic components of a vertebrate-like anticipatory immune system does not preclude a functional equivalent in invertebrates [13], and there exists evidence of enhanced secondary responses to homologous infectious challenges [14-22]. Moreover, in invertebrates, the genetic background of both hosts and parasites plays a critical role in determining the probability of infection, a phenomenon called genetic specificity [13,23,24]. Thus invertebrate defences do not lack sophistication, but the genetic and cellular mechanisms that underlie either invertebrate genetic specificity or enhanced secondary responses remain obscure (but see [25]).

Alternative splicing could permit a single gene to mediate alternative immune responses via the production of multiple proteins, and the flexibility of such a mechanism could have important implications for the spread of resistance alleles [26]. The Down syndrome cell adhesion molecule (Dscam), which can take some tens of thousands of different forms through alternative splicing, is commonly associated with its function in the vertebrate and invertebrate nervous systems, but seems to also play a role in invertebrate immunity [27-30]. In the fruit fly *Drosophila*, *Dscam *is expressed in cell types that play major roles in the fly's immune system, and RNA interference-mediated depletion of *Dscam *was shown to impair the insect's capacity to engulf bacteria by phagocytosis [29]. Similarly, the silencing of *Anopheles gambiae Dscam *(*AgDscam*) compromises the mosquito's ability to resist *Plasmodium *[30]. Moreover, AgDscam produces pathogen-specific splice form repertoires upon immune challenge [30]. In particular, the Dscam repertoire in response to parasite exposure differs between bacteria and *Plasmodium *and between *Plasmodium berghei *and *Plasmodium falciparum *[30]. However, such specificity has so far only been observed in studies comparing these divergent *Plasmodium *parasites, which probably last shared a common ancestor around 55 million years ago [31].

The response of *AgDscam *transcription to *P. falciparum *diversity (i.e. within-species rather than between-species parasite exposure) may shed light on the resolution of the innate immune system's specificity and dynamics, as well as its limitations. In theory, 31,920 unique splice forms of Dscam can be generated through the alternative splicing of 84 variable exons contained within three variable exon cassettes (these are exon 4, exon 6, and exon 10) [30], and which could potentially contribute to a capability to distinguish between different genotypes of *Plasmodium*. This capability would imply a more specific innate immune response than previously supposed. Here, research is described that relates *P. falciparum *genetic diversity to the expression characteristics of the alternatively spliced Dscam receptor in the *An. gambiae *mosquito.

Two independent experiments were performed. The first was a field study that utilized freshly harvested blood from human subjects for which the genetic diversity of naturally acquired *P. falciparum *infections was characterized. The second experiment was based in the laboratory, where mosquitoes were exposed to either single parasite clones or mixtures of clones contained within human red blood cells in culture following an established protocol [32]. AgDscam receptor diversity was studied in two ways. First, a diversity index was calculated based on exon 4 and exon 6 frequencies to assess whether overall *AgDscam *expression diversity increased under exposure to *P. falciparum *parasites, and if it increased further with greater parasite infection diversity. Second, it was assessed whether particular *Dscam *exon transcripts were associated with infection diversities (in the field study) or particular parasite genotypes (in the laboratory study).

## Methods

### Mosquito infection in Kenya

Blood was obtained from primary school students in Iguhu (34°45'E, 0°10'N) in Kakamega district, western Kenya. The predominant malaria vector species in the area is *An. gambiae s.s. *[33,34]. During and shortly after the rainy season, children (5-14 years of age) were screened for gametocytes by thick blood-films stained in Giemsa's stain.

Gametocyte carriers who had >40 gametocytes/μL of blood and who consented to participate in the study were asked to donate 10 mL of blood, which was obtained intravenously by a clinician, and drawn into heparinized tubes. A total of six gametocyte donors were used in this study (two donors per gametocyte-positive infection group). Most of this blood was used for mosquito infections through membrane feeders, with around 50 μL also spotted onto Whatman paper for later DNA extraction using a Chelex-100 isolation technique [35]. Methods for infecting mosquitoes are described in [36]. Drawn blood was immediately centrifuged at 700 × *g*, and the serum discarded and replaced with human AB serum (Cambrex Bio Science, Walkersville, MD, USA). Blood was then placed in warmed membrane feeders. Five to seven-day old *Anopheles gambiae *Kisumu strain mosquitoes were placed in paper cups at a density of 60/cup and allowed to feed on the infected blood for 30 minutes. Mosquitoes used in this experiment were originally obtained near the Kenya Medical Research Institute in Kisumu [37], but had been bred in an insectary and adapted to feed from a membrane feeder for many years (thus the mosquitoes were unlikely to be highly polymorphic).

A total of twenty four mosquitoes were used in the field study (three mosquitoes per treatment × four infection groups × two independent replicates). Mosquitoes were transferred to cages post-blood meal, and then placed into RNAlater (Ambion) at 24-hours post-blood meal. Mosquitoes were harvested 24-hours post-feeding because this is the peak time that *Plasmodium *ookinetes penetrate the mosquito midgut [30]. Total RNA extraction was carried out using a Qiagen RNeasy Mini kit. On day 7 after they had been exposed to infected blood, the remaining fed mosquitoes from each cage were dissected in 2% mercurochrome and examined for oocysts to confirm the presence of *Plasmodium *infections.

### *Plasmodium *microsatellite typing

To estimate *P. falciparum *diversity, microsatellite loci were chosen based on their strength in terms of percentage PCR positives, frequency distributions of allele length important for sizing amplicons [38], size in base-pairs [39], and allele frequencies [40]. A hemi-nested PCR reaction was carried out to amplify six select microsatellite loci from each DNA extraction sample [38]. Applied Biosystems Genemapper v4.0 software was used to automate the measurement of allele length and to quantify peaks in samples containing multiple alleles per locus. Only peaks from samples that amplified >200 fluorescent units were included in the deduction of infection diversity. Multiple alleles per locus were scored if the minor microsatellite peaks were >33% the height of the predominant allele.

### Mosquito infection in the laboratory

Five to seven-day old female *An. gambiae *(Keele strain) mosquitoes were offered blood meals containing *in vitro *grown gametocytes of two different genotypes of *P. falciparum *(clone 3D7 [41] and clone HB3 [42]) through membrane feeders, and three whole mosquitoes per treatment were harvested after 24-hours. Three independent experiments on three different dates were performed. A total of thirty six mosquitoes were used in this study (three mosquitoes per treatment × four treatment groups × three independent replicates). The gametocyte culture and membrane feeding protocols followed those previously described [32]. The isolation of total RNA was carried out using a Qiagen RNeasy Mini technology kit.

### Quantifying Dscam diversity

RNA extracted from three individual mosquitoes per treatment was reverse transcribed using random hexamers and primers were designed to amplify a fragment of *AgDscam *spanning from exon 3 to exon 7 (primer sequences were: Forward; 5' - GTATACGCCTGCATGGCTAAGA - 3', Reverse; 5' - GCCCTTATCCTCCTTCTTG - 3'). Thus, the amplicons comprised variable exons 4 and 6, and the conserved exon 5. PCR products were cloned using a TOPO TA cloning kit with pCR4-TOPO vector and transformed in chemically competent *Escherichia coli*. Up to 48 clones per mosquito were sequenced in a 96-capillary ABI 3730*xl *DNA Analyzer (provided by the Gene Pool Sequencing facility, University of Edinburgh). Sequences were aligned using BioEdit version 7.0.9.0 and identified by cross-referencing with the known *An. gambiae *genome sequence available from the Ensembl genome browser.

### Statistical analyses

Diversity was measured with Simpson's Index (1-D) [43], which quantified the combination of expressed exon 4 and exon 6 variants in each transcript as determined by sequencing. The presence and abundance of each individual exon, and exon 4:6 combination was identified. Statistical analysis of data using general linear modelling and one-way analysis of variance was carried out using Minitab 15.1.1.0 software. For the laboratory study, 'clone' was the single fixed effect with three levels, 3D7, HB3 or mixture of both, and for the field study, the number of genotypes detected (single, double or triple infection) was a fixed effect and donor was added as a random effect to account for variation. Data were normalized with a square root transformation of Simpson's Index (D) before analyses. The Neighbour-Joining trees (additional file 1) were produced using PHYLIP and variation in counts of exon groupings were carried out with chi-square contingency table analysis.

### Ethical statement

The ethical review boards of the Kenya Medical Research Institute, Kenya reviewed and approved our protocol for screening of *P. falciparum *gametocyte carriers and subsequent intravenous blood drawing. Written, informed consent to participate in the study was provided by all study subjects and/or their parents or guardians (see also [36]). All gametocyte donors were subsequently treated with amodiaquine by the clinicians at the Iguhu Health Centre, per the guideline of the Ministry of Health of Kenya. Feeding of mosquitoes was conducted in a secure, insect-proof room at the Iguhu Health Centre.

## Results and Discussion

For the field study, *An. gambiae *were membrane-fed on blood samples taken from gametocyte-carrying children, and RNA was harvested from the insects 24-hours post-exposure, a time when parasites are traversing the midgut epithelium [30]. cDNAs were then cloned and sequenced to identify specific *AgDscam *gene variants (at exons 4 and 6) expressed within insects fed from different blood samples. Control blood was taken from children carrying no *Plasmodium *(as detected with microscopy). A set of *P. falciparum *microsatellite markers [38] were used to identify parasite genotypes in gametocyte carriers. As blood samples contain whole populations of parasites, it is not possible to precisely estimate the number of genotypes present. However, by simply counting alleles at each locus, it is possible to identify the minimum number of genotypes present in a sample, and thus the methods used yielded a lower-bound of parasite genetic diversity. Single, double and triple infections were subsequently identified.

**Figure 1:**
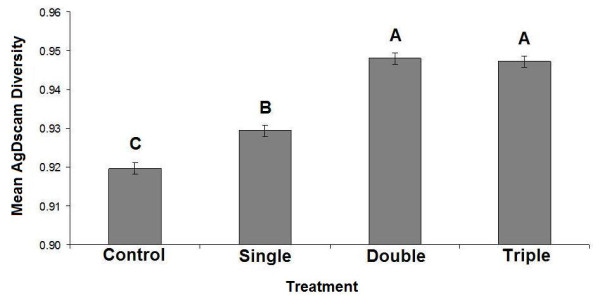
***Anopheles gambiae Dscam *expression diversity versus increasing parasite diversity in the field**. AgDscam diversity (as 1- *Simpson's Index*) increased between controls and exposed treatments and between single and double genotype-exposures only. Levels not connected by same letter are significantly different. Error bars represent standard error (SE). **Key**: Control; blood meal with no parasites, Single; mosquitoes exposed to a minimum of one *P. falciparum *genotype, Double; mosquitoes exposed to a minimum of two *P. falciparum *genotypes, Triple; mosquitoes exposed to a minimum of three *P. falciparum *genotypes.

*AgDscam *expression, as characterized by a diversity index [43] and averaged over both studied exons, was affected by the blood that the mosquitoes fed upon in the field (F_3,20 _= 3.22, P = 0.045; Figure 1). It appears that AgDscam is more diverse when parasites are present (diversity was significantly lower in uninfected blood controls than in all other samples; Figure 1). It is the combined diversity at exons 4 and 6 that drive this pattern, as a relationship between expression diversity and parasite diversity was not apparent when the exons were studied separately (exon 4: F_1,22 _= 3.33, P = 0.081; exon 6: F_1,22 _= 0.02, P = 0.901). It was interesting to note that there was a non-statistically significant trend for an association between exon 4 (but not exon 6) diversity and parasite diversity. This may imply that exon 4 has a bigger role than exon 6 in responding to *Plasmodium *in the field. Additionally, AgDscam diversity was also higher when mosquitoes fed upon blood with double infections when compared to single infections, but diversity did not rise further with triple infections (Figure 1). Thus, these field data provided only limited evidence of a link between host response recognition capacity and parasite intraspecific diversity. As the blood stage diversity as measured by microsatellites could not be certain to be representative of gametocyte diversity, i.e. it is conceivable that not all parasite types were producing gametocytes (see [44]), a laboratory-based study was carried out using gametocyte-producing lines of *P. falciparum*. In this way, the diversity of gametocytes entering the experimental mosquitoes could be certain.

**Figure 2:**
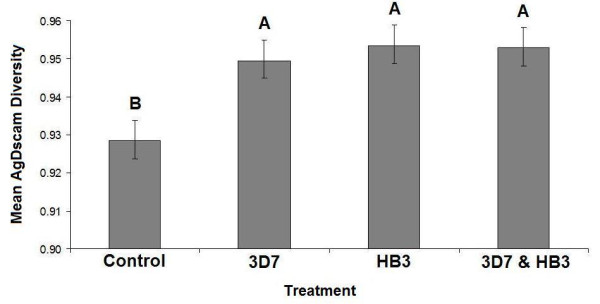
***Anopheles gambiae Dscam *expression diversity versus increasing parasite diversity in the lab**. AgDscam diversity (as 1- *Simpson's Index*) increased between controls and exposed treatments, but did not increase between the single genotype-exposure and the mixed genotype-exposure. Levels not connected by same letter are significantly different. Error bars represent standard error (SE). **Key**: Control; blood meal with no parasites, 3D7; mosquitoes exposed to *Plasmodium falciparum *clone 3D7, HB3; mosquitoes exposed to *Plasmodium falciparum *clone HB3, 3D7 & HB3; mosquitoes exposed to both clones in the same blood meal.

For the laboratory study, mosquitoes were membrane-fed on blood infected with gametocytes of either *P. falciparum *clone 3D7 [41] or HB3 [42], or a mixture of the two, and RNA was harvested from the insects 24-hours post-exposure. cDNAs were cloned and sequenced and *AgDscam *exon 4 and exon 6 variants expressed were identified within mosquitoes exposed to different treatments (control blood with no *Plasmodium*, clone 3D7, clone HB3, and clones 3D7 and HB3 mixed). It was found that *AgDscam *expression was affected by the blood that the mosquitoes fed upon (F_3,32 _= 5.29, P = 0.004; Figure 2). Thus, as with the field study, it appears that AgDscam is more diverse when parasites are present (diversity was significantly lower in uninfected blood controls than in all other samples; Figure 2). Although diversity increased under exposure to either laboratory parasite clone, it did not increase further in response to a mixture of the two clones (Figure 2). As with the field data, it was the combined diversity at exons 4 and 6 that drove this pattern, as variation in Dscam diversity was not apparent when the exons were studied separately (exon 4: F_1,34 _= 3.10, P = 0.087; exon 6: F_1,34 _= 0.16, P = 0.690). Again, there was some evidence of a trend for exon 4 but not exon 6, as previously seen in the field study, implying that the variability rendered by exon 4 in responding to *Plasmodium *may be relatively more important than the variability offered by exon 6 expression.

A χ^2 ^analysis was used to identify whether particular splice variants were over- or under-represented in any of the field (control, single, double or triple) or laboratory (control, clone 3D7, clone HB3, mixed) infections. Because the extreme splice variation leads to very low counts for individual exons, exon variants were grouped into categories based on their genetic distance. Although this grouping may not reflect any relationship between biological function and antigen recognition similarity, varying the cut-off points did not change the qualitative results. Based on a Neighbour-Joining (NJ) tree (exon 4, see additional file 1; exon 6, see additional file 1), genetic groupings of exon variants were defined such that exon 4 variants were clustered into 3 groups (see additional file 1), and exon 6 into 3 groups also (see additional file 1).

χ^2 ^contingency tests were used to determine whether the number of observations (counts) of each group differed between the infection categories (see additional file 2 for raw data). In general, no exons were over- or under-represented in any particular infection grouping in either the field or laboratory experiments. The only exceptions to this were exon 4 variants 4.11, 4.12 and 4.13 which are clearly a distinct genetic grouping (see additional file 1), and were under-represented in control mosquitoes compared to exposed treatments in the field (Pearson's Chi-Square = 6.318, DF = 2, P = 0.042), and exon 6 variants 6.1 and 6.9 which are also a distinct genetic grouping (see additional file 1), and were under-represented in control mosquitoes, but also over-represented in mosquitoes exposed to a single genotype of *P. falciparum *in the field (Pearson Chi-Square = 19.975, DF = 6, P-Value = 0.003). These data suggests that exon 4 variants 4.11, 4.12 and 4.13, and exon 6 variants 6.1 and 6.9, may be particularly important for the mosquito's immune response to *Plasmodium *in the field.

## Conclusion

The results show an increase in AgDscam splice-form diversity at 24-hours post-exposure, when the parasites are crossing the insect's midgut epithelium [30,45,46]. This observation, confirmed both in the field and the laboratory, reinforce that the AgDscam receptor responds to *Plasmodium *at a vital stage for the parasite's development. The limited association between AgDscam diversity and *P. falciparum *genotype-diversity in the field raises the possibility that the alternatively-spliced receptor could be responding to *P. falciparum *diversity. This observation, however, was not seen between double- and triple-exposed treatments. Although it cannot be assumed that every parasite genotype detected in the blood samples in the field is represented in the sexual stage, there is an apparent consistency in the results showing a lack of genotype-specific *Dscam *expression diversity following two different experimental approaches (field and laboratory). Naturally, the field data may also be affected by unmeasured factors, for example some of the blood samples could conceivably have harboured other parasite species. This possibility was investigated using PCR detection for other *Plasmodium *parasites (specifically *Plasmodium malariae, Plasmodium ovale *and *Plasmodium vivax*), and the presence of *P. malariae *in both of the blood samples containing a single genotype of *P. falciparum *was recorded (see additional file 3). Consequently, the single infections were confounded with multi-species infections, and yet AgDscam diversity of these multi-species exposures was lower than the diversity levels of the double and triple *P. falciparum *exposures. Although even higher *Dscam *expression diversity could be expected in this instance, this was not observed, and thus other interpretations are possible depending on whether one species has a stronger influence on *Dscam *expression within a particular multiple-infection than another. It was also interesting to note that the over-representation of the group of exon 6 variants (variants 6.1 and 6.9) found in these multi-species infections was not found in the single-species infections, implying that these variants could be influenced by the presence of *P. malariae*. The significance of these observations remains to be investigated. It was also determined whether *P. falciparum *infection intensity in the blood samples, i.e. abundance of parasite stages (as determined by quantitative PCR), could have affected our results, but we found no relationship between parasite abundance and Dscam diversity (see additional file 3). Finally, future studies may benefit from the use of multiple mosquito genotypes. It is possible that different genotypes may respond differently to the same parasite challenge in terms of *AgDscam *expression. This may be a logical direction for future work as the relative absence of related studies on AgDscam allows little speculation on whether the colonies used in this study would be a good proxy for what can be expected in natural populations. Thus, in summary, the data clearly indicate that AgDscam diversity increases with parasite exposure, but they do not suggest that AgDscam diversity rises further in response to increased parasite diversity.

## Competing interests

The authors declare that they have no competing interests.

## Authors' contributions

TJL, YAA and GY conceived the experiment. TJL, GY and LRC designed the experiment. PHS, JMM and YAA carried out the experiment. PHS, DJO and TJL analysed the data. PHS and TJL wrote the paper. All authors read, commented on, and approved the final manuscript.

## Supplementary Material

Additional file 1 Additional information containing phylogenetic trees and contingency tests. Click here for file

Additional file 2 Additional file containing exon 4 and exon 6 raw data. Click here for file

Additional file 3 Details of additional experiments. Click here for file
