# Supplementary material for: Alternative splicing of the Anopheles gambiae Dscam gene in diverse Plasmodium falciparum infections
Source: Malar J. 2011 Jun 8;10:156. doi: 10.1186/1475-2875-10-156 (PMC3118162; doi:10.1186/1475-2875-10-156)
Supplement: Additional file 1 — Additional information containing phylogenetic trees and contingency tests. [file 1475-2875-10-156-S1.PDF]

Alternative splicing of the *Anopheles gambiae Dscam* gene in diverse *Plasmodium falciparum* infections

Additional File 1

Neighbour-Joining tree construction and chi-square contingency table analysis

Methods

Full *AgDscam* exon variant DNA sequences were aligned using ClustalW and Neighbour-Joining tree constructed with MEGA (version 4) software.

Results

Figure S1: Neighbour-Joining tree of *AgDscam* exon 4 variants clustered into 3 groups. Full-length DNA sequences of the 14 variants of exon 4 were aligned using BioEdit software version 7.0.9.0, and the tree constructed using the MEGA (version 4) program.

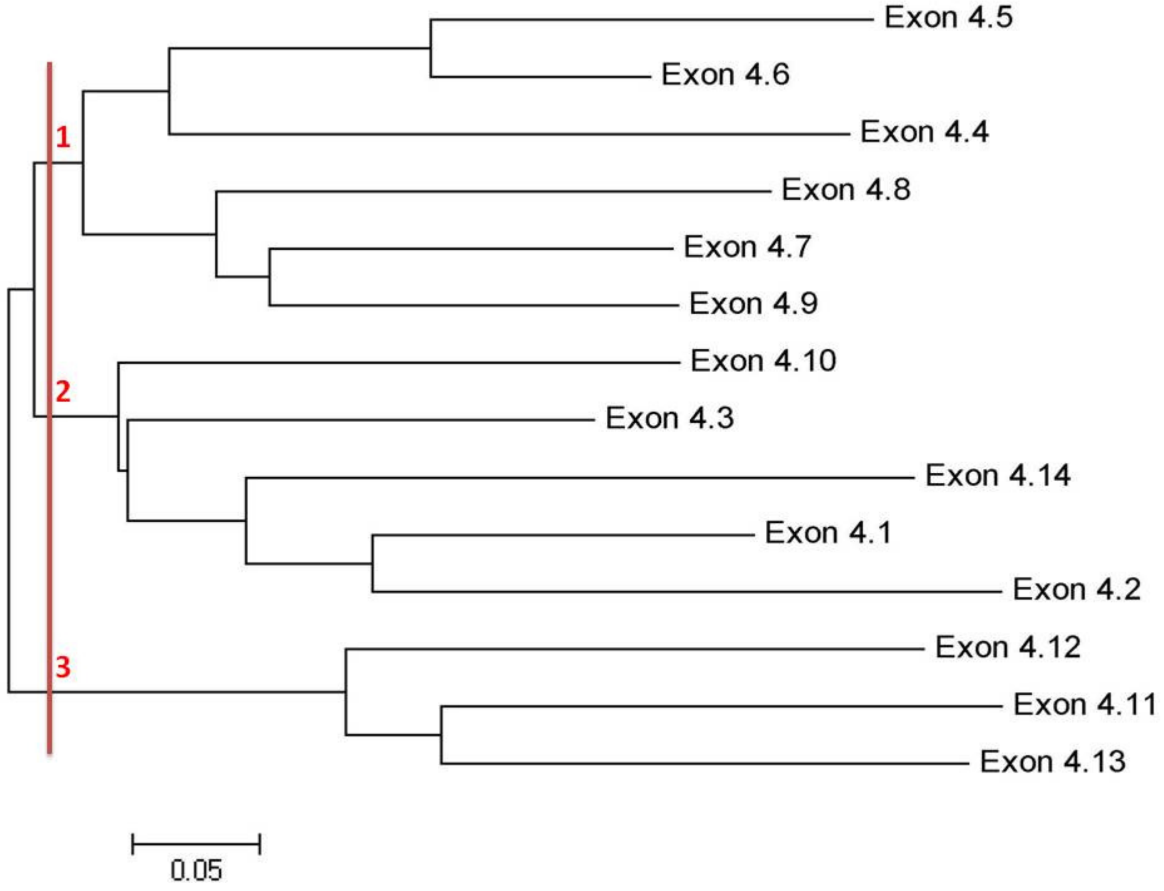

Table S1a: Cross tabulation and chi-square results for exon 4 clustered into 3 groups in our NJ tree between control and exposed treatments in the *field*. Analysis was carried out using Minitab® 15.1.1.0. Exon 4 variants 4.11, 4.12 and 4.13 are represented as Group 3 and were under-represented in control mosquitoes compared to exposed treatments in the field (Pearson Chi-Square = 6.318, DF = 2, P-Value = 0.042).

**Treatment**		**Count**		
		Group 1	***Group 2***	***Group 3***
Control	Observed	82	59	12
	Expected	79.7	52.3	21.0
Exposed	Observed	255	162	77
	Expected	257.3	168.7	68.0

Table S1b: Cross tabulation and chi-square results for exon 4 clustered into 3 groups in our NJ tree between controls, single-, double- and triple-exposed treatments in the *field*. Analysis was carried out using Minitab® 15.1.1.0. Differences between observed and expected values were not statistically significant (Pearson Chi-Square = 8.970, DF = 6, P-Value = 0.175).

**Treatment**		**Count**		
		Group 1	***Group 2***	***Group 3***
Control	Observed	82	59	12
	Expected	79.7	52.3	21.1
Single	Observed	82	51	18
	Expected	78.7	51.6	20.8
Double	Observed	94	63	33
	Expected	99.0	64.9	26.1
Triple	Observed	79	48	26
	Expected	79.7	52.3	21.1

Table S1c: Cross tabulation and chi-square results for exon 4 clustered into 3 groups in our NJ tree between control and exposed treatments in the *lab*. Analysis was carried out using Minitab® 15.1.1.0. Differences between observed and expected values were not statistically significant (Pearson Chi-square = 3.231, DF = 2, P-Value = 0.199).

**Treatment**		**Count**		
		Group 1	***Group 2***	***Group 3***
Control	Observed	169	108	43
	Expected	159.3	121.6	39.0
Exposed	Observed	558	447	135
	Expected	567.7	433.4	139.0

Table S1d: Cross tabulation and chi-square results for exon 4 clustered into 3 groups in our NJ tree between controls, 3D7-, HB3- and mixed exposed treatments in the *lab*. Analysis was carried out using Minitab® 15.1.1.0. Differences between observed and expected values were not statistically significant (Pearson Chi-Square = 4.724, DF = 6, P-Value = 0.580).

**Treatment**		**Count**		
		Group 1	***Group 2***	***Group 3***
Control	Observed	169	108	43
	Expected	159.3	121.6	39.0
3D7	Observed	193	148	42
	Expected	190.7	145.6	46.7
HB3	Observed	173	144	49
	Expected	182.2	139.1	44.6
3D7/HB3	Observed	192	155	44
	Expected	194.7	148.6	47.7

Table S1e: Cross tabulation and chi-square results for exon 4 clustered into 3 groups in our NJ tree between 3D7 and HB3 treatments in the *lab*. Analysis was carried out using Minitab® 15.1.1.0. Differences between observed and expected values were not statistically significant (Pearson Chi-Square = 1.301, DF = 2, P-Value = 0.522).

**Treatment**		**Count**		
		Group 1	***Group 2***	***Group 3***
3D7	Observed	193	148	42
	Expected	187.2	149.3	46.5
HB3	Observed	173	144	49
	Expected	178.8	142.7	44.5

Figure S2: Neighbour-Joining tree of *AgDscam* exon 6 variants clustered into 3 groups. Full-length DNA sequences of 28 variants of exon 6 were aligned using BioEdit software version 7.0.9.0, and the tree constructed using the MEGA (version 4) program.

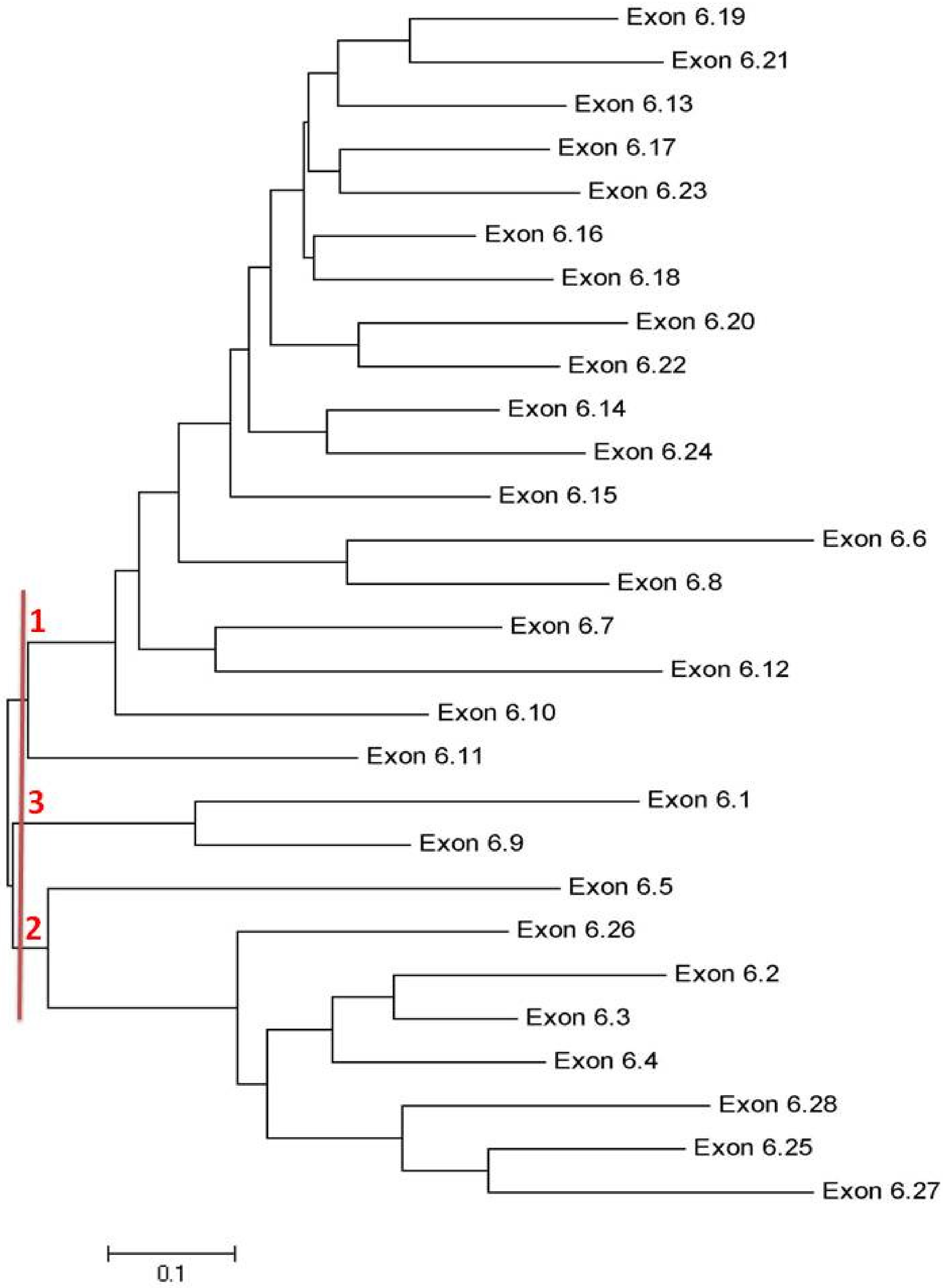

Table S2a: Cross tabulation and chi-square results for exon 6 clustered into 3 groups in our NJ tree between control and exposed treatments in the *field*. Analysis was carried out using Minitab® 15.1.1.0. Differences between observed and expected values were not statistically significant (Pearson Chi-Square = 5.243, DF = 2, P-Value = 0.073).

**Treatment**		**Count**		
		Group 1	***Group 2***	***Group 3***
Control	Observed	105	29	19
	Expected	94.6	30.4	27.9
Exposed	Observed	315	106	105
	Expected	325.4	104.6	96.1

Table S2b: Cross tabulation and chi-square results for exon 6 clustered into 3 groups in our NJ tree between controls, single-, double- and triple-exposed treatments in the *field*. Analysis was carried out using Minitab® 15.1.1.0. Exon 6 variants 6.1 and 6.9 are represented as Group 3 and appeared to be under-represented in control mosquitoes but over-represented in mosquitoes exposed to a single parasite genotype in the field (Pearson Chi-Square = 19.975, DF = 6, P-Value = 0.003).

**Treatment**		**Count**		
		Group 1	***Group 2***	***Group 3***
Control	Observed	105	29	19
	Expected	94.6	30.4	27.9
Single	Observed	87	25	47
	Expected	98.4	31.6	29.0
Double	Observed	122	43	31
	Expected	121.2	39.0	35.8
Triple	Observed	106	38	27
	Expected	105.8	34.0	31.2

Table S2c: Cross tabulation and chi-square results for exon 6 clustered into 3 groups in our NJ tree between control and exposed treatments in the *lab*. Analysis was carried out using Minitab® 15.1.1.0. Differences between observed and expected values were not statistically significant (Pearson Chi-Square = 1.446, DF = 2, P-Value = 0.485).

**Treatment**		**Count**		
		Group 1	***Group 2***	***Group 3***
Control	Observed	182	56	68
	Expected	172.8	60.4	72.8
Exposed	Observed	599	217	261
	Expected	608.2	212.6	256.2

Table S2d: Cross tabulation and chi-square results for exon 6 clustered into 3 groups in our NJ tree between controls, 3D7, HB3 and mixed exposures in the *lab*. Analysis was carried out using Minitab® 15.1.1.0. Differences between observed and expected values were not statistically significant (Pearson Chi-Square = 3.984, DF = 6, P-Value = 0.679).

**Treatment**		**Count**		
		Group 1	***Group 2***	***Group 3***
Control	Observed	182	56	68
	Expected	172.8	60.4	72.8
3D7	Observed	209	64	90
	Expected	205.0	71.6	86.4
HB3	Observed	189	71	79
	Expected	191.4	66.9	80.6
Mix	Observed	201	82	92
	Expected	211.8	74.0	89.2

Table S2e: Cross tabulation and chi-square results for exon 6 clustered into 3 groups in our NJ tree between 3D7 and HB3 exposures in the *lab*. Analysis was carried out using Minitab® 15.1.1.0. Differences between observed and expected values were not statistically significant (Pearson Chi-Square = 1.265, DF = 2, P-Value = 0.531).

**Treatment**		**Count**		
		Group 1	***Group 2***	***Group 3***
3D7	Observed	209	64	90
	Expected	205.8	69.8	87.4
HB3	Observed	189	71	79
	Expected	192.2	65.2	81.6
