# Supplementary material for: Alternative splicing of the Anopheles gambiae Dscam gene in diverse Plasmodium falciparum infections
Source: Malar J. 2011 Jun 8;10:156. doi: 10.1186/1475-2875-10-156 (PMC3118162; doi:10.1186/1475-2875-10-156)
Supplement: Additional file 2 — Additional file containing exon 4 and exon 6 raw data. [file 1475-2875-10-156-S2.PDF]

Additional File 2: *AgDscam* individual exon variant counts

Experiment	Feed	Treatment	Replicate	Exon 6 variant																											
				6.1	*6.2*	*6.3*	*6.4*	*6.5*	*6.6*	*6.7*	*6.8*	*6.9*	*6.10*	*6.11*	*6.12*	*6.13*	*6.14*	*6.15*	*6.16*	*6.17*	*6.18*	*6.19*	*6.20*	*6.21*	*6.22*	*6.23*	*6.24*	*6.25*	*6.26*	*6.27*	*6.28*
**Field**	1	SH158 +++	1	0	0	1	2	1	0	4	0	0	0	4	1	0	1	0	0	1	1	0	0	1	0	1	0	1	2	0	2
		SH158 +++	2	4	0	3	0	0	1	0	1	7	2	0	0	0	0	2	0	2	0	4	0	3	0	1	2	0	0	3	1
		SH158 +++	3	0	0	0	1	4	1	0	3	1	1	0	0	2	0	2	0	2	2	4	0	0	0	0	3	0	1	0	0
		SH79 ++	1	2	0	2	0	0	0	0	5	2	0	4	1	0	0	0	0	1	3	0	2	1	0	1	5	0	7	0	0
		SH79 ++	2	7	0	0	1	1	0	2	2	0	1	0	1	6	0	0	1	1	1	0	1	0	4	0	0	0	2	0	0
		SH79 ++	3	5	0	1	2	4	1	3	0	0	3	1	1	0	1	0	1	1	2	0	0	0	1	4	1	0	2	0	3
		K10 +	1	7	0	0	0	0	1	0	0	1	0	0	0	3	0	0	2	0	0	1	0	1	0	1	1	0	4	2	0
		K10 +	2	2	0	2	0	1	0	0	0	0	0	0	0	0	0	2	0	1	3	1	0	5	1	3	1	0	0	0	0
		K10 +	3	9	0	0	3	0	0	0	1	3	3	0	0	3	2	1	1	1	0	1	2	0	0	2	4	0	2	0	0
		SH165 -	1	4	1	0	1	2	0	1	4	0	2	0	0	0	0	1	1	0	1	2	6	3	0	0	0	1	0	0	1
		SH165 -	2	5	0	0	0	5	0	0	3	0	0	1	0	4	2	0	2	0	0	1	1	1	0	1	7	0	0	0	0
		SH165 -	3	3	1	1	1	0	1	0	2	0	1	0	0	0	0	4	0	0	1	0	1	5	0	3	0	0	0	0	0
	2	SH93 +++	1	4	0	1	0	2	0	2	6	2	1	0	0	1	0	1	0	0	0	2	0	1	1	1	4	0	0	3	0
		SH93 +++	2	2	0	0	2	2	1	0	1	2	1	3	1	0	0	2	0	1	1	2	0	1	0	0	2	0	1	0	1
		SH93 +++	3	1	0	1	0	1	1	2	1	4	0	1	0	1	0	0	0	4	2	3	0	2	0	0	1	0	1	0	1
		SH25 ++	1	4	0	0	2	0	1	3	0	2	2	1	1	2	1	0	1	1	3	0	1	0	0	1	1	0	1	0	0
		SH25 ++	2	2	0	0	0	2	0	1	2	0	1	1	1	1	5	0	3	3	0	0	1	3	2	1	1	0	1	0	2
		SH25 ++	3	7	0	0	0	3	0	0	3	0	1	2	1	0	0	0	0	1	0	0	0	0	3	1	2	0	7	0	0
		IG14 +	1	15	1	0	0	1	0	0	0	0	0	0	0	0	1	0	4	2	1	0	1	1	0	0	0	0	0	0	1
		IG14 +	2	1	1	0	0	0	0	0	0	1	2	2	0	1	1	1	4	2	0	2	0	0	0	0	0	0	0	1	2
		IG14 +	3	8	0	0	1	0	0	0	3	0	0	1	0	0	2	1	1	0	3	0	0	0	0	1	2	0	3	0	0
		SH82 -	1	2	1	1	0	1	0	0	2	0	2	2	1	0	1	0	1	1	1	0	1	0	2	0	0	0	2	1	0
		SH82 -	2	2	0	0	0	2	0	0	0	0	0	0	1	0	0	1	0	1	4	3	0	0	0	1	0	0	2	0	0
		SH82 -	3	2	0	0	0	1	3	4	3	1	0	1	0	2	0	0	0	0	0	1	0	2	2	0	0	0	4	0	0
**Lab**	1	Unfed Con	1	12	0	0	2	0	0	1	3	0	0	1	0	4	0	0	0	2	0	0	0	0	0	0	2	0	0	1	0
		Unfed Con	2	14	1	0	1	0	1	0	3	0	3	0	1	0	0	0	0	0	2	3	0	1	2	0	0	0	0	0	0
		Unfed Con	3	23	0	0	3	1	2	0	7	0	2	3	2	0	2	0	0	0	0	0	1	0	0	0	0	0	0	0	0
		FedCon	1	11	0	2	0	0	1	0	0	0	1	1	0	2	1	0	7	0	6	2	1	1	0	5	1	0	4	0	0
		FedCon	2	8	0	4	0	0	0	1	0	2	0	0	2	3	0	1	1	0	2	0	1	1	2	0	0	0	4	2	0
		FedCon	3	6	0	3	0	0	0	2	0	1	1	1	0	5	3	0	2	0	1	0	0	0	0	0	3	0	2	2	0
		3D7	1	8	0	5	0	2	1	0	5	0	2	0	3	2	0	0	0	1	4	1	1	0	2	3	0	0	1	2	2
		3D7	2	7	0	1	1	0	1	1	2	1	5	0	1	1	1	2	4	0	5	1	3	1	1	0	1	0	3	0	0
		3D7	3	15	2	3	1	1	2	0	3	0	0	1	0	2	3	0	2	0	0	0	0	3	3	0	0	0	0	0	1
		HB3	5	13	0	4	0	2	1	0	2	0	0	4	0	0	1	2	0	0	2	3	2	0	1	1	2	1	2	0	1
		HB3	2	3	1	3	0	0	0	2	0	0	0	1	1	2	0	0	1	0	2	2	1	1	3	0	0	0	4	0	2
		HB3	3	13	0	1	0	2	1	1	0	2	1	1	1	2	0	3	0	0	3	0	2	2	0	1	0	0	2	0	4
		MIX	1	7	0	3	2	0	1	1	2	2	1	0	0	0	1	0	2	0	4	0	3	1	1	2	2	0	4	1	0
		MIX	2	5	0	3	0	5	3	1	1	2	0	1	0	1	1	4	1	0	2	2	0	1	2	2	1	0	3	0	0
		MIX	3	14	0	2	0	1	3	0	1	0	0	3	1	0	0	0	0	1	3	1	0	1	2	1	0	0	1	0	0
	2	FedCon	1	9	0	2	2	0	3	1	0	0	0	3	0	0	2	0	3	3	1	2	0	0	3	0	2	0	1	1	0
		FedCon	2	4	0	1	0	2	1	2	17	1	1	0	1	3	0	1	1	2	1	1	1	2	1	0	2	0	2	1	0
		FedCon	3	2	0	1	0	1	2	0	0	0	1	0	0	2	0	1	0	0	5	2	0	1	1	0	2	0	3	0	0
		3D7	1	4	0	6	1	1	1	0	8	4	0	1	0	0	0	0	1	2	3	1	1	1	2	0	1	0	0	0	0
		3D7	2	6	1	3	0	0	0	1	1	1	0	1	2	2	2	0	0	1	0	1	1	1	1	1	0	0	2	0	1
		3D7	3	19	1	2	0	1	2	1	3	0	2	1	0	0	0	0	0	1	7	0	3	0	0	1	1	0	0	1	0
		HB3	1	2	0	1	0	0	1	2	3	1	2	0	0	1	1	0	0	1	1	2	1	4	0	2	1	0	0	0	0
		HB3	2	9	0	1	1	1	1	2	7	0	1	0	1	5	2	1	1	1	4	2	1	0	1	0	0	0	0	0	1
		HB3	3	17	1	1	0	1	0	0	3	0	0	0	0	1	1	0	0	5	5	1	1	0	3	1	1	0	0	3	2
		MIX	1	2	0	3	0	6	4	1	2	1	1	1	0	4	0	2	0	0	2	2	1	4	2	1	0	1	2	0	5
		MIX	2	11	0	1	1	2	0	0	0	0	2	0	0	2	1	0	1	1	4	1	0	0	0	3	0	0	2	0	0
		MIX	3	10	0	3	2	0	5	0	5	0	1	1	1	3	0	0	0	0	2	3	0	0	1	0	0	0	0	3	0
	3	FedCon	1	16	0	1	0	2	2	0	2	0	0	4	2	1	0	2	2	2	0	0	2	1	0	0	1	0	1	4	0
		FedCon	2	0	0	0	3	0	0	4	1	1	0	0	2	0	0	1	0	2	0	0	3	0	1	2	0	0	0	2	0
		FedCon	3	3	0	2	0	1	0	2	1	4	0	0	0	0	0	0	0	0	1	1	0	0	0	1	1	0	0	0	0
		3D7	1	13	0	0	0	0	1	3	7	1	1	0	0	0	0	2	0	0	0	4	1	0	2	1	0	0	1	0	0
		3D7	2	3	0	3	0	0	2	2	4	2	1	2	0	3	0	0	3	0	2	0	2	0	4	0	1	0	6	0	0
		3D7	3	4	5	0	0	2	1	2	0	2	2	1	3	2	0	0	0	1	0	7	2	4	0	3	0	0	1	0	1
		HB3	1	6	0	0	3	2	4	1	2	1	2	1	1	0	1	1	0	1	3	6	1	0	4	0	0	0	3	3	0
		HB3	2	10	1	2	2	0	2	0	1	1	0	1	1	2	2	0	1	0	0	3	0	0	1	1	0	0	1	1	2
		HB3	3	1	3	3	2	0	2	0	1	0	2	0	0	0	0	1	0	0	1	0	3	2	4	1	0	0	1	0	0
		MIX	1	7	0	5	0	3	0	1	14	2	2	1	0	1	0	0	1	0	1	1	1	2	0	2	2	0	0	1	1
		MIX	2	1	0	5	0	1	8	0	1	4	0	0	2	1	0	1	3	3	3	4	0	0	4	0	2	0	0	0	1
		MIX	3	23	0	3	1	1	3	0	2	1	0	1	1	0	2	1	0	0	2	0	0	0	1	0	2	0	2	2	0

Experiment	Feed	Treatment	Replicate	Exon 4 variant													
				4.1	*4.2*	*4.3*	*4.4*	*4.5*	*4.6*	*4.7*	*4.8*	*4.9*	*4.10*	*4.11*	*4.12*	*4.13*	*4.14*
**Field**	1	SH158 +++	1	5	0	0	0	3	2	0	0	3	0	1	0	0	0
		SH158 +++	2	4	1	1	0	3	5	7	2	0	2	3	1	1	1
		SH158 +++	3	3	0	5	0	3	6	3	0	0	0	1	1	4	1
		SH79 ++	1	3	1	0	0	5	7	2	8	0	0	0	1	0	5
		SH79 ++	2	5	0	1	4	7	2	0	6	0	2	1	2	1	4
		SH79 ++	3	5	1	3	3	1	1	1	2	1	1	6	2	3	3
		K10 +	1	3	2	1	0	3	3	1	4	0	0	1	1	2	1
		K10 +	2	0	7	0	2	1	3	4	0	1	0	2	0	0	1
		K10 +	3	2	2	3	0	4	6	3	7	2	0	0	0	2	5
		SH165 -	1	0	0	1	0	3	3	7	2	2	0	0	0	0	4
		SH165 -	2	5	2	0	3	9	6	4	0	0	0	1	0	0	4
		SH165 -	3	15	0	0	0	2	2	3	0	0	1	0	1	0	0
	2	SH93 +++	1	1	0	3	1	8	4	2	4	0	1	1	0	4	3
		SH93 +++	2	6	0	1	0	1	3	4	0	0	1	2	2	2	0
		SH93 +++	3	5	1	2	0	1	6	5	0	3	0	2	0	1	1
		SH25 ++	1	5	0	3	3	6	1	0	5	0	0	2	2	1	2
		SH25 ++	2	8	0	0	1	5	4	1	1	0	1	4	2	2	2
		SH25 ++	3	2	0	1	0	3	3	1	10	0	0	1	1	2	5
		IG14 +	1	4	1	0	0	2	4	6	6	0	0	1	0	1	6
		IG14 +	2	3	0	0	0	4	9	0	0	0	0	1	0	1	4
		IG14 +	3	2	3	1	2	1	1	3	0	0	0	4	0	2	0
		SH82 -	1	6	1	2	2	1	5	3	1	0	2	1	0	2	2
		SH82 -	2	5	0	0	0	2	2	3	0	1	0	0	0	1	3
		SH82 -	3	2	0	0	0	7	4	1	1	3	0	5	1	0	4
**Lab**		Unfed Con	1	4	3	0	2	7	6	6	7	0	0	2	0	2	6
		Unfed Con	2	6	1	0	0	1	1	1	5	2	0	0	3	4	8
		Unfed Con	3	3	1	2	5	7	5	6	4	2	1	4	1	2	5
	1	FedCon	1	12	0	0	5	3	5	6	0	1	1	1	1	7	5
		FedCon	2	4	1	0	3	6	4	5	1	2	0	8	0	1	1
		FedCon	3	8	0	0	0	0	5	6	2	0	0	0	0	3	8
		3D7	1	14	0	0	3	7	5	1	6	3	0	1	1	2	3
		3D7	2	8	0	0	1	8	0	7	2	0	3	0	1	4	9
		3D7	3	12	1	0	0	5	5	3	5	0	4	0	0	5	7
		HB3	5	7	0	0	1	6	6	5	5	0	1	2	1	4	9
		HB3	2	15	0	0	0	1	3	2	8	3	0	0	0	1	3
		HB3	3	8	0	0	0	4	8	7	3	0	0	0	1	8	5
		MIX	1	4	0	1	0	7	6	5	5	0	1	4	2	1	8
		MIX	2	10	0	1	0	1	4	5	3	0	0	0	0	1	15
		MIX	3	8	2	0	2	6	0	8	3	0	2	5	0	1	3
	2	FedCon	1	8	0	0	0	7	6	3	7	3	0	1	1	3	6
		FedCon	2	4	2	0	2	2	2	17	6	0	0	1	0	3	5
		FedCon	3	3	0	0	2	2	6	5	2	0	0	0	0	3	5
		3D7	1	5	1	0	4	4	6	7	2	0	2	0	0	2	6
		3D7	2	3	0	3	1	3	6	5	2	0	1	3	1	1	2
		3D7	3	12	0	0	2	14	2	4	4	0	0	4	0	4	2
		HB3	1	4	2	0	1	2	1	4	0	0	0	0	0	2	9
		HB3	2	14	0	0	1	3	4	5	4	0	0	1	0	4	11
		HB3	3	4	1	0	0	12	0	10	3	0	1	0	0	8	6
		MIX	1	7	0	3	1	3	9	3	4	1	1	0	0	4	11
		MIX	2	6	0	1	5	4	2	3	4	0	0	3	0	3	4
		MIX	3	7	1	1	1	1	5	9	9	1	2	0	1	4	5
	3	FedCon	1	11	0	0	0	12	3	7	3	0	3	0	0	6	3
		FedCon	2	0	1	0	0	5	4	0	1	1	0	4	0	0	6
		FedCon	3	3	0	1	0	4	2	0	0	1	0	0	0	0	7
		3D7	1	6	0	0	3	4	2	8	3	1	0	2	0	3	7
		3D7	2	6	3	1	3	9	0	4	6	2	0	0	0	3	8
		3D7	3	11	0	0	0	7	5	4	5	0	2	4	0	1	6
		HB3	1	6	0	0	7	7	8	3	3	3	1	1	1	3	5
		HB3	2	7	0	0	3	3	6	2	3	1	0	4	1	4	3
		HB3	3	11	3	2	0	4	3	2	3	0	1	0	1	2	5
		MIX	1	4	1	0	2	5	5	9	3	0	3	4	0	3	6
		MIX	2	9	0	0	4	4	3	5	5	1	0	2	0	0	13
		MIX	3	8	0	0	6	4	9	4	3	0	0	4	1	1	7
