# Supplementary material for: Alternative splicing of the Anopheles gambiae Dscam gene in diverse Plasmodium falciparum infections
Source: Malar J. 2011 Jun 8;10:156. doi: 10.1186/1475-2875-10-156 (PMC3118162; doi:10.1186/1475-2875-10-156)
Supplement: Additional file 3 — Details of additional experiments. [file 1475-2875-10-156-S3.PDF]

Alternative splicing of the *Anopheles gambiae Dscam* gene in diverse *Plasmodium falciparum* infections

Additional File 3

PCR detection of Plasmodium species

Methods

Existing primers for *Plasmodium falciparum, P. malariae, P. ovale* and *P. vivax* 18s rRNA, and nested PCR conditions were taken from [1]. Template DNA was extracted from blood samples spotted onto Whatman® paper using a Chelex-100 isolation technique [2].

Results

All blood samples were infected with *P. falciparum*, no blood was infected with *P. ovale* or *P. vivax*, while two samples contained *P. malariae* in addition to *P. falciparum* (Table S3).

Table S3: Scoring of presence or absence of four species of *Plasmodium* parasite following PCR detection.

Treatment	*P. falciparum*	*P. malariae*	*P.ovale*	*P.vivax*
SH158 (triple infection_#1)	+	-	-	-
SH93 (triple infection_#2)	+	-	-	-
SH79 (double infection_#1)	+	-	-	-
SH25 (double infection_#2)	+	-	-	-
K10(single infection_#1)	+	+	-	-
IG14 (single infection_#2)	+	+	-	-

Key: +; Present, -; Absent

Plasmodium falciparum parasite intensity

Methods

Real-time quantitative PCR assays were performed on an Applied Biosystems StepOnePlus cycler and we used the non-specific nucleic acid stain SYBR Green I which preferentially binds to dsDNA. For our reference gene, human-specific Glyceraldehyde 3-phosphate dehydrogenase (GAPDH) primers were designed using Primer3 software and checked for quality (tendency to dimerise, cross-dimerise, hairpin etc) with NetPrimer (primers were: F: 5' – CGACCACTTTGTCAAGCTCA – 3', R: 5' - GGTGGTCCAGGGGTCTTACT – 3') and amplified an 112bp fragment. Existing primers were used to amplify *Plasmodium falciparum* SSU rRNA (primers were: F: 5' – TCTAGGGGAACTATTTTAGCTT – 3', R: 5' – CACAGTAAATGCTTTAACTGTT – 3') isolating an 180bp fragment [3]. All cycler runs included serial dilutions of parasite DNA to indicate primer efficiency and included a melting curve analysis for product identification. Each reaction contained: 8µl SYBR Green Master Mix, 10µl ddH_2O, 1µl primer mix (10mM concentration) and 1µl template genomic DNA. PCR conditions were:- holding stage: 95ºC for 10 minutes; cycling stage: 40 cycles of 95ºC for 15 seconds, 55ºC for 60 seconds; and melt curve stage: 95ºC for 15 seconds, 55ºC for 60 seconds, temperature increment +0.3ºC, 95ºC for 15 seconds.

We quantified relative parasite intensity for two blood samples which were confirmed (using microsatellite loci sizing) to contain a minimum of one *P. falciparum* genotype (K10, IG14; single infections), two blood samples containing a minimum of two *P. falciparum* genotypes (SH79, SH25; double infections), and two blood samples containing a minimum of three *P. falciparum* genotypes (SH158, SH93; triple infections). Mean C_t values were calculated from 3 replicates per treatment. Delta C_t values were calculated (Delta C_t = Mean $C_{t,sample}$ – Mean $C_{t,reference}$), and we plotted fold difference between target DNA and reference DNA ($2^{-\text{delta Ct}}$) (Figure S3).

Results

Figure S3: Fold difference in relative DNA quantity between human reference (GAPDH) and target (*Plasmodium falciparum*).

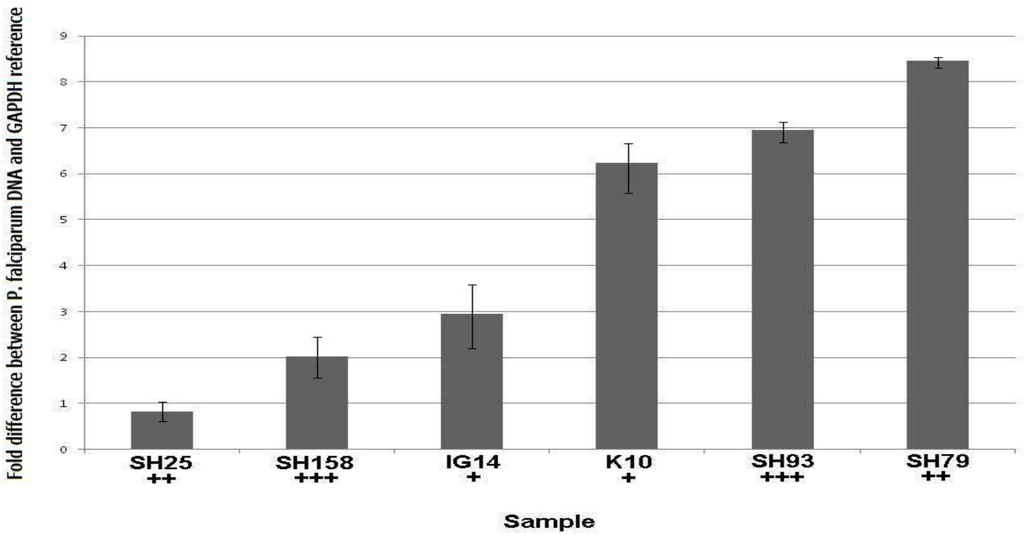

Key: +++; triple infection, ++; double infection, +; single infection.

Acknowledgements

We thank Lydia Chambers for carrying out the *Plasmodium falciparum* PCR detection assay.

References

1. Snounou G, Viriyakosol S, Zhu XP, Jarra W, Pinheiro L, Dorosario VE, Thaithong S, Brown KN: **High-sensitivity of detection of human malaria parasites by the use of nested polymerase chain-reaction**. *Molecular and Biochemical Parasitology* 1993, **61**(2):315-320.
2. Wooden J, Kyes S, Sibley CH: **PCR and Strain Identification in *Plasmodium falciparum***. *Parasitology Today* 1993, **9**(8):303-305.
3. Bell AS, Ranford-Cartwright LC: **A real-time PCR assay for quantifying Plasmodium falciparum infections in the mosquito vector**. *International Journal for Parasitology* 2004, **34**(7):795-802.
